# Supplementary material for: Impact of Heat Stress on Meat Quality and Antioxidant Markers in Iberian Pigs
Source: Antioxidants (Basel). 2021 Nov 29;10(12):1911. doi: 10.3390/antiox10121911 (PMC8750345; doi:10.3390/antiox10121911)
Supplement: Supplementary file 1 [file antioxidants-10-01911-s001.zip › antioxidants-1454378-supplementary.pdf]

Tabla S1. Pearson correlation between the variables studied. First value: r; second value: *P*. NS: non-significant.

	DM	Fat	Ash	Prot	Kcal	Fe	Zn	pH_{24h}	L*	a*	b*	C*	h°	$C_{18:1}$	SFA	MU<br>FA	PUFA	MDA	ABTS	DP<br>PH	FRAP	GPx	CAT
DM		0.872<br>0.0001	-0.579<br>0.0001	NS	0.969<br>0.0001	NS	0.300<br>0.0383	NS	-0.344<br>0.017	0.432<br>0.0021	0.561<br>0.0001	0.462<br>0.0009	NS	NS	NS	NS	NS	NS	0.4420<br>0.0017	NS	0.3551<br>0.0133	NS	-0.335<br>0.0201
Fat	0.872<br>0.0001		-0.629<br>0.0001	NS	0.950<br>0.0001	NS	NS	NS	NS	NS	0.407<br>0.0041	NS	0.335<br>0.0198	NS	NS	NS	NS	NS	NS	NS	NS	NS	NS
Ash	-0.579<br>0.0001	-0.629<br>0.0001		NS	-0.599<br>0.0001	NS	NS	NS	0.290<br>0.0455	NS	NS	NS	NS	NS	NS	NS	NS	NS	-0.321<br>0.0262	NS	NS	NS	NS
Prot	NS	NS	NS		NS	NS	NS	-0.366<br>0.0105	NS	NS	NS	0.323<br>0.0251	-0.308<br>0.0332	NS	NS	NS	NS	NS	0.383<br>0.0071	NS	0.403<br>0.0045	NS	-0.385<br>0.0070
Kcal	0.969<br>0.0001	0.950<br>0.0001	-0.599<br>0.0001	NS		NS	0.335<br>0.0201	NS	-0.286<br>0.0484	0.319<br>0.0273	0.507<br>0.0002	0.362<br>0.0114	NS	NS	NS	NS	NS	NS	0.359<br>0.0123	NS	NS	NS	NS
Fe	NS	NS	NS	NS	NS		0.329<br>0.0224	NS	-0.442<br>0.0017	0.630<br>0.000	0.537<br>0.0001	0.630<br>0.0001	NS	0.361<br>0.0116	-0.290<br>0.0454	0.291<br>0.0448	NS	0.445<br>0.0015	NS	NS	NS	-391<br>0.0060	-0.459<br>0.0010
Zn	0.300<br>0.0383	0.277<br>0.0566	NS	NS	0.335<br>0.0201	0.329<br>0.0224		0.397<br>0.0052	NS	NS	0.425<br>0.0026	NS	0.372<br>0.0093	NS	NS	NS	NS	NS	NS	NS	NS	NS	NS
pH_{24h}	NS	NS	NS	-0.366<br>0.0105	NS	NS	0.397<br>0.0052		-0.079<br>0.5919	NS	NS	NS	0.384<br>0.007	NS	0.336<br>0.0194	-0.295<br>0.0418	NS	NS	NS	NS	-0.293<br>0.0433	NS	NS
L*	-0.344<br>0.0168	NS	0.290<br>0.0455	NS	-0.286<br>0.0484	-0.442<br>0.0017	NS	NS		-0.694<br>0.0001	-0.602<br>0.0001	-0.696<br>0.0001	NS	NS	NS	NS	-0.381<br>0.0075	-0.476<br>0.0007	-0.388<br>0.0065	-0.350<br>0.0147	NS	0.386<br>0.0068	0.375<br>0.0086
a*	0.432<br>0.0021	NS	NS	0.362<br>0.0114	0.319<br>0.0273	0.630<br>0.0001	NS	NS	-0.694<br>0.0001		0.830<br>0.0001	0.989<br>0.0001	NS	NS	-0.319<br>0.0272	NS	0.378<br>0.0080	0.424<br>0.0026	0.419<br>0.0031	NS	0.330<br>0.0207	-0.412<br>0.0036	-0.592<br>0.0001
b*	0.561<br>0.0001	0.4066<br>0.0041	NS	NS	0.507<br>0.0002	0.537<br>0.0001	0.4254<br>0.0026	NS	-0.602<br>0.0001	0.8299<br>0.0001		0.8845<br>0.0001	0.4343<br>0.0020	NS	NS	NS	NS	0.467<br>0.0008	0.441<br>0.0017	NS	NS	-0.405<br>0.0043	-0.557<br>0.0001
C*	0,462<br>0.0009	NS	NS	0,323<br>0.0251	0.362<br>0.0114	0.630<br>0.0001	NS	NS	-0.696<br>0.0001	0.989<br>0.0001	0.8845<br>0.0001		NS	NS	NS	NS	0.325<br>0.0243	0.441<br>0.0017	0.422<br>0.0028	NS	0.305<br>0.0350	-0.440<br>0.0018	-0.625<br>0.0001
h°	NS	0,335<br>0.0198	NS	-0,308<br>0.0332	NS	NS	0.372<br>0.0093	0.3842<br>0.0070	NS	NS	0.434<br>0.0020	NS		NS	0.389<br>0.0063	NS	NS	NS	NS	NS	NS	NS	NS
$C_{18:1}$	NS	NS	NS	NS	NS	0.3614<br>0.0116	NS	NS	NS	NS	NS	NS	NS		-0.638<br>0.0001	0.936<br>0.0001	-0.547<br>0.0001	NS	NS	NS	NS	NS	NS
SFA	NS	0,274<br>0.0597	NS	NS	NS	-0.290<br>0.0454	NS	0.336<br>0.0194	NS	-0.319<br>0.0272	NS	NS	0.389<br>0.0063	-0.638<br>0.0001		-0.707<br>0.0001	NS	NS	NS	NS	-0.336<br>0.0196	NS	NS
MU<br>FA	NS	NS	NS	NS	NS	0.291<br>0.0448	NS	-0.295<br>0.0418	NS	NS	NS	NS	NS	0.936<br>0.0001	-0.707<br>0.0001		-0.554<br>0.0001	NS	NS	NS	NS	NS	NS
PUFA	-NS	NS	NS	NS	NS	NS	NS	NS	-0.381<br>0.0075	0.378<br>0.0080	NS	0.325<br>0.0243	NS	-0.547<br>0.001	NS	-0.554<br>0.0001		NS	NS	NS	NS	NS	NS
MDA	NS	NS	NS	NS	NS	0.445<br>0.0015	NS	NS	-0.475<br>0.0007	0.424<br>0.0026	0.467<br>0.0008	0.441<br>0.0017	NS	NS	NS	NS	NS		0.418<br>0.0031	NS	NS	NS	NS
ABTS	0.442	NS	-0.321	0.383	0.359	NS	NS	NS	-0.388	0.419	0.441	0.422	NS	NS	NS	NS	NS	0.418		NS	NS	NS	-0.293

	0.0017		0.0262	0.0071	0.0123				0.0065	0.0031	0.0017	0.0028						0.0031					0.0432
DP PH	NS	NS	NS	NS	NS	NS	NS	NS	-0.350<br>0.0147	NS	NS	NS	NS	NS	NS	NS	NS	NS	NS		NS	NS	NS
FRAP	0.355<br>0.0133	NS	NS	0.403<br>0.0045	NS	NS	NS	-0.293<br>0.0433	NS	0.333<br>0.0207	NS	0.305<br>0.0350	NS	NS	-0.336<br>0.0196	NS	NS	NS	NS	NS		NS	NS
GPx	NS	NS	NS	NS	NS	-0.391<br>0.0060	NS	NS	0.386<br>0.0068	-0.412<br>0.0036	-0.405<br>0.0043	-0.440<br>0.0018	NS	NS	NS	NS	NS	NS	NS	NS	NS		0.327<br>0.0235
CAT	-0.335<br>0.0201	NS	NS	-0.385<br>0.0070	NS	-0.459<br>0.0010	NS	NS	0.375<br>0.0086	-0.592<br>0.0001	-0.557<br>0.0001	-0.625<br>0.0001	NS	NS	NS	NS	NS	NS	-0.293<br>0.0432	NS	NS	0.327<br>0.0235	
